# GLP-1RA may have varying effects on cardiac structure in patients with ASCVD depending on BMI

**DOI:** 10.3389/fendo.2024.1355540

**Published:** 2024-03-15

**Authors:** Ling Xu, Dan Zhu

**Affiliations:** Department of Cardiology, Peking University Third Hospital, NHC Key Laboratory of Cardiovascular Molecular Biology and Regulatory Peptides, Peking University, Beijing, China

**Keywords:** GLP-1RA, cardiac remodeling, body mass index, weight loss, ASCVD

## Abstract

**Background:**

Glucagon-like peptide-1 receptor agonist(GLP-1RA) is commonly used in patients with cardiovascular disease due to its significant improvement in the prognosis of atherosclerotic cardiovascular disease (ASCVD). However, previous studies have primarily focused on obese patients, leaving uncertainty regarding whether GLP-1RA can yield similar cardiovascular benefits in individuals with normal or low body weight.

**Methods:**

In this study, we enrolled patients with ASCVD to establish a retrospective cohort. Patients receiving GLP-1RA treatment were assigned to the GLP-1RA group, while a control group was formed by matching age and body mass index (BMI) among patients not receiving GLP-1RA treatment. Each group was further divided into subgroups based on baseline BMI levels: normal weight, overweight, and obesity. A six-month follow-up was conducted to assess changes in patient weight, metabolic indicators, and cardiac structure and function.

**Results:**

Among the normal weight subgroup, no significant weight change was observed after six months of GLP-1RA treatment (57.4 ± 4.8 vs. 58.7 ± 9.2, p = 0.063). However, significant weight reduction was observed in the other two subgroups (Overweight group: 70.0 ± 9.1 vs. 73.1 ± 8.2, p = 0.003, Obesity group: 90.5 ± 14.3 vs. 95.5 ± 16.6, p<0.001). Regardless of baseline BMI levels, GLP-1RA demonstrated significant glucose-lowering effects in terms of metabolic indicators. However, GLP-1RA have a more significant effect on improving blood lipids in overweight and obese patients. The effects of GLP-1RA on cardiac structure exhibited variations among patients with different baseline BMI levels. Specifically, it was observed that the improvement in atrial structure was more prominent in patients with normal body weight(LAD: 33.0 (30.3, 35.5) vs. 35.0 (32.5, 37.1), p = 0.018, LAA (18.0 (16.0, 21.5) vs. 18.5 (16.5, 20.5), p = 0.008), while the enhancement in ventricular structure was more significant in obese subjects(LEVDD: 49.8 ± 5.8 vs. 50.2 ± 5.0, p < 0.001, LVMI: 65.1 (56.2, 71.4) vs. 65.8 (58.9, 80.4), p < 0.039).

**Conclusion:**

According to the study, it was found that the administration of GLP-1RA can have different effects on cardiac structure in patients with different baseline BMI, In obese patients, improvements in ventricular remodeling may be more associated with weight loss mechanisms, while in patients with normal or low BMI, GLP-1RA may directly improve atrial remodeling through GLP-1 receptors in atrial tissue.

## 1 Introduction

Nowadays, there is an urgent need to identify optimal treatment strategies for atherosclerotic cardiovascular disease (ASCVD) due to its high morbidity and mortality. Recent cardiovascular outcome trials (CVOTs) have provided evidence that the Glucagon-like peptide-1 receptor agonist (GLP-1RA) is a promising hypoglycemic drug that can delay the progression of atherosclerosis (1, 2) and improve the prognosis of ASCVD patients (3–5). However, GLP-1RA is commonly recommended for obese patients due to its favorable effect on weight loss. Previous CVOTs primarily involved obese individuals, resulting in a relatively small representation of Asian subjects with lower baseline body weight (3, 4, 6, 7). A meta-analysis has indicated that a body mass index(BMI) below 28.4kg/m^2^ is associated with an increased risk of cardiovascular death as BMI decreases (8). Hence, it is essential to investigate whether GLP-1RA can provide cardiac benefits across all BMI groups. In light of this, a retrospective cohort study was conducted to assess the impact of GLP-1RA on cardiac structure and function in ASCVD patients from various BMI groups. The findings of this study aim to provide valuable insights for the clinical application of GLP-1RA in treating ASCVD.

## 2 Methods

According to the local ethical standards of the Medical Science Research Ethics Committee, and in compliance with the 1964 Helsinki Declaration and its subsequent amendments, all procedures were conducted and all experimental protocols were approved by the Medical Science Research Ethics Committee (M2023228). Since this study was a retrospective cohort study that only gathered clinical data from participants, an exemption from informed consent was obtained after a review by the Medical Science Research Ethics Committee. There was no formal involvement of patients or the public in the design or implementation of this trial.

This retrospective cohort study enrolled patients diagnosed with ASCVD and treated with GLP-1RA at the Cardiology Department of Peking University Third Hospital between January 2021 and April 2022. A control group was then matched with ASCVD patients who did not receive GLP-1RA based on age and BMI. The participants were categorized into three groups according to their BMI levels: normal weight group (BMI < 24kg/m^2^), overweight group (24 ≤ BMI < 28 kg/m^2^), and obesity group (BMI ≥ 28 kg/m^2^). Baseline information and clinical data were retrospectively collected at 3 months and 6 months after enrollment for subsequent statistical analysis.

### 2.1 Participants

Our study enrolled patients aged 18 years or older who had been diagnosed with ASCVD, including coronary heart disease(CHD), stroke, and peripheral artery atherosclerosis, at the Cardiology Department of Peking University Third Hospital between January 2021 and April 2022. Patients who were receiving GLP-1RA treatment were included in the GLP-1RA group, while patients who were not receiving GLP-1RA were included in the control group after matching for age and BMI.

The exclusion criteria for our study were as follows: 1) Acute coronary syndrome and unstable heart failure; 2) Acute complications of diabetes mellitus, such as diabetic ketoacidosis, hyperosmolar coma, and hypoglycemia; 3) Other organic heart diseases besides CHD, such as congenital heart disease, cardiomyopathy, myocarditis, valvular heart disease, and pulmonary heart disease; 4) End-stage malignancy; 5) Pregnancy and lactation; and 6) Lack of clinical data or medication switching during follow-up.

### 2.2 Data and clinical outcomes

Demographic information and laboratory examinations were collected retrospectively at baseline, as well as at the 3rd and 6th month of follow-up. Echocardiographic parameters were also obtained at baseline and the 6th month of follow-up. All data were extracted from computerized medical records by clinical staff. The study’s primary endpoint was to evaluate the variations in cardiac structural and functional parameters among different BMI groups during the follow-up period.

### 2.3 Statistical analysis

Categorical data were presented as numbers and percentages and were compared using either the chi-square test or Fisher’s exact test. The distribution of all continuous variables was assessed using P-P plots. Non-normally distributed continuous variables were described using the median and interquartile range and analyzed using the Mann-Whitney U test. Normally distributed continuous variables were described as the mean ± standard deviation and analyzed using independent t-tests. Changes in normally distributed variables over the follow-up period were evaluated using paired t-tests or repeated measures analysis of variance, depending on the number of follow-up visits. Changes in non-normally distributed variables during the follow-up were assessed using the Friedman test or the Wilcoxon rank-sum test. Furthermore, univariate and multivariate analyses, employing generalized estimating equations, were conducted to investigate whether the use of GLP-1RA was an independent factor associated with cardiac structural and functional changes. All statistical analyses were performed using SPSS Statistics for Windows, Version 26.0 (Armonk, NY: IBM Corp), and Stata statistical software, release 13 (StataCorp). Two-sided statistical tests were used, and a significance level of p < 0.05 was considered statistically significant.

## 3 Results

### 3.1 Baseline information

In the comparison between the two groups, it was observed that the control group had a higher proportion of male patients (79.3%) compared to the GLP-1RA group (66.7%). In terms of comorbidities, the GLP-1RA group had a higher proportion of patients with hyperlipidemia and diabetes, while the proportion of patients with CHD and stroke was lower. Additionally, the GLP-1RA group had a higher prevalence of SGLT2i use and worse basal glucose levels, while statin use was more common in the control group. However, there were no significant differences in cardiac structure and function between the two groups (refer to **Table 1** for detailed information).

**Table 1:** Baseline information of the two groups.

	GLP-1RA (N=140)	Control (N=140)	P Value
General Condition			
** Gender[Male, N (%)]**	92 (66.7)	111 (79.3)	0.016
** Age (y)**	56.0 (44.0, 65.0)	58.0 (48.0, 67.0)	0.106
** Height (cm)**	169.5 (163.0, 174.0)	170.0 (165.0, 174.0)	0.503
** Weight (kg)**	83.3 ± 18.5	81.5 ± 15.5	0.354
** Waist (cm)**	101.0 (92.0, 109.0)	98.0 (97.0, 106.0)	0.058
** Hip Circumference (cm)**	106.0 (100.0, 112.0)	105.0 (100.8, 111.0)	0.600
Past Medical History			
** Hypertension, N (%)**	104 (74.8)	98 (70.0)	0.661
** Hyperlipidemia, N (%)**	102 (72.9)	82 (58.6)	0.017
** Diabetes Mellitus, N (%)**	133 (95.0)	70 (50.0)	<0.001
** CHD,N (%)**	69 (49.2)	117 (83.6)	<0.001
** Stroke,N (%)**	6 (4.3)	22 (15.7)	0.002
** Heart Failure,N (%)**	4 (2.9)	2 (1.4)	0.447
** CKD,N (%)**	1 (0.7)	0 (0)	0.498
Medications			
** GLP-1RA, N (%)**	140 (100.0)	0 (0)	<0.001
** **Liraglutide,N (%)	105 (75.0)	0 (0)	–
** **Dulaglutide,N (%)	31 (22.1)	0 (0)	–
** **Semaglutide,N (%)	4 (2.9)	0 (0)	–
** SGLT2i,N (%)**	46 (32.9)	12 (8.6)	<0.001
** Other OADs, N (%)**	76 (54.7)	37 (26.8)	<0.001
** Insulin, N (%)**	25 (18.0)	10 (7.2)	0.007
** AECI/ARB/ARNI,N (%)**	78 (55.7)	84 (60.0)	0.545
** Statins,N (%)**	99 (70.7)	123 (88.5)	<0.001
** β-blocker,N (%)**	76 (54.3)	86 (61.9)	0.226
** CCBs,N (%)**	50 (35.7)	46 (33.1)	0.706
Baseline Biomakers (Serum sample)			
** FBG (mmol/L)**	7.2 (6.5, 9.4)	5.9 (5.2, 7.6)	<0.001
** HbA1c (%)**	7.4 (6.6, 8.8)	6.2 (5.9, 7.2)	<0.001
** Uric acid (umol/L)**	370.6 ± 103.7	357.8 ± 101.9	0.449
** LDL-c (mmol/l)**	2.5 (1.8, 3.2)	2.5 (1.9, 3.1)	0.893
** NT-proBNP (pg/dl)**	125.0 (42.2, 409.3)	122.0 (55.5, 431.5)	0.334
Echocardiography			
** Left atrial diameter (mm)**	36.2 (34.0, 38.8)	36.0 (33.2, 38.9)	0.379
** Left atrial area (cm^2^)**	19.0 (17.0, 22.0)	20.0 (18.2, 22.4)	0.248
** LVESD (mm)**	30.0 (28.0, 32.0)	30.0 (28.0, 34.0)	0.174
** LVEDD (mm)**	49.5 ± 4.8	48.9 ± 7.6	0.499
** LVMI (g/cm^2^)**	67.3 (58.8, 81.3)	70.9 (61.9, 81.0)	0.168
** LVEF (%)**	69.0 (65.0, 72.0)	68.0 (63.0, 72.0)	0.307
** E/A**	0.8 (0.7, 1.0)	0.9 (0.7, 1.1)	0.056
** E/Em**	7.0 (6.0, 8.0)	7.0 (6.0, 8.0)	0.456

Normally distributed data are expressed as the mean ± standard deviation, and nonnormally distributed data are expressed as the median (interquartile range).

CHD, coronary heart disease; CKD, chronic kidney disease; GLP-1RA , Glucagon-like peptide-1 receptor agonist; OADs, Oral hypoglycemic drugs; ACEI, angiotensin converting enzyme inhibitor; ARB, angiotensin receptor blocker; ARNI, angiotensin receptor-neprilysin inhibitor; SGLT2i, sodium-glucose cotransporter 2 inhibitors; CCBs, calcium channel blockers; FBG, fasting blood glucose; LDL-c, low-density lipoprotein cholesterol; LVESD, left ventricular end-systolic diameter; LVEDD, left ventricular end-diastolic diameter; LVMI, left ventricular mass index; LVEF, left ventricular ejection fraction.

Based on the analysis of the data, we observed variations in indicators among different BMI groups. As BMI increases, the subjects tend to be younger, and the proportion of CHD is lower. In the group receiving GLP-1RA, there is also a decrease in the proportion of stroke, whereas this is not observed in the control group. When examining cardiac structure, we found that an increase in BMI is associated with an increase in left atrial diameter (LAD), left atrial area (LAA), and left ventricular end-diastolic diameter (LVEDD). However, there were no significant differences in cardiac function among the different BMI groups (refer to **Table 2** for detailed information).

**Table 2:** Baseline information of patients in different BMI groups.

	GLP-1RA (N=140)					Control (N=140)				
	BMI<24kg/m2 (N=22)	BMI 24-28kg/m2 (N=35)		BMI≥28kg/m2 (N=83)	P Value	BMI<24kg/m2 (N=22)	BMI 24-28kg/m2 (N=50)		BMI≥28kg/m2 (N=68)	P Value
General Condition										
** Gender[Male, N (%)]**	9 (40.9)	22 (62.9)	61 (73.5)		0.012	16 (72.7)	43 (86.0)		52 (76.5)	0.320
** Age (y)**	64.5 (61.0, 72.0)	58.0 (50.0, 66.0)	51.0 (40.0, 63.0)		<0.001	66.5 (60.5, 71.0)	57.5 (51.0, 67.3)		55.0 (43.5, 66.0)	<0.001
** Height (cm)**	162.5 (156.8, 169.3)	169.0 (163.0, 172.0)	170.0 (165.0, 175.0)		0.005	168.0 (163.8, 172.5)	170.0 (165.0, 174.3)		170.0 (163.0, 175.0)	0.546
** Weight (kg)**	58.7 ± 9.2	73.1 ± 8.2	94.2 ± 14.2		<0.001	64.2 ± 6.7	73.8 ± 6.7		92.7 ± 13.5	<0.001
** Waist (cm)**	86.5 (84.0, 91.3)	95.0 (90.0, 98.0)	107.0 (102.0, 115.0)		<0.001	85.5 (83.0, 91.0)	95.0 (91.0, 99.0)		106.0 (103.0, 111.0)	<0.001
** Hip Circumference (cm)**	97.0 (94.8, 99.3)	100.0 (97.0, 106.0)	110.5 (106.0, 116.3)		<0.001	99.3 (96.0, 102.0)	104.0 (100.0, 106.0)		110.5 (105.8, 116.0)	<0.001
Past Medical History										
** Hypertension, N (%)**	13 (59.1)	28 (80.0)	63 (75.9)		0.340	11 (50.0)	32 (64.0)	55 (80.9)		<0.001
** Hyperlipidemia, N (%)**	16 (72.7)	23 (65.7)	63 (75.9)		0.524	12 (54.5)	29 (58.0)	41 (60.3)		0.888
** Diabetes Mellitus, N (%)**	22 (100.0)	34 (97.1)	77 (92.8)		0.307	9 (40.9)	20 (40.0)	41 (60.3)		0.061
** CHD,N (%)**	14 (63.6)	20 (57.1)	35 (42.2)		0.091	21 (95.5)	48 (98.0)	48 (70.6)		<0.001
** Stroke,N (%)**	3 (13.6)	2 (5.7)	1 (1.2)		0.035	1 (4.5)	11 (22.0)	10 (14.7)		0.164
** Heart Failure,N (%)**	1 (4.5)	1 (2.9)	2 (2.4)		0.871	0 (0)	1 (2.0)	1 (1.5)		0.804
** CKD,N (%)**	0 (0)	0 (0)	1 (1.2)		0.705	0 (0)	0 (0)	0 (0)		–
Medications										
** AECI/ARB/ARNI,N (%)**	8 (36.4)	22 (62.9)	48 (57.8)		0.122	8 (36.4)	31 (62.0)	45 (66.2)		0.043
** SGLT2i,N (%)**	8 (36.4)	15 (42.9)	23 (27.7)		0.259	2 (9.1)	4 (8.0)	6 (8.8)		0.980
** Statins,N (%)**	17 (77.3)	29 (82.9)	53 (63.9)		0.089	21 (95.5)	47 (94.0)	55 (80.9)		0.073
** β-blocker,N (%)**	8 (36.4)	19 (54.3)	49 (59.0)		0.165	12 (54.5)	32 (64.0)	42 (61.8)		0.735
** CCBs,N (%)**	6 (27.3)	13 (37.1)	31 (37.3)		0.667	5 (22.7)	14 (28.0)	27 (39.7)		0.199
Baseline Biomakers (Serum sample)										
** FPG (mmol/L)**	7.1 (6.0, 9.7)	7.1 (6.7, 9.9)	7.4 (6.1, 11.2)		0.879	5.8 (5.0, 9.0)	5.7 (5.0, 7.9)	6.1 (5.4, 7.3)		0.777
** HbA1c (%)**	8.3 (6.6, 10.2)	7.2 (6.9, 7.9)	7.6 (6.5, 8.9)		0.659	6.2 (5.8, 7.1)	6.2 (5.7, 7.3)	6.2 (5.8, 7.2)		0.996
** Uric acid (umol/L)**	329.2 ± 75.7	359.9 ± 92.9	384.7 ± 111.3		0.090	309.9 ± 78.6	349.4 ± 90.9	384.0 ± 111.8		0.011
** LDL-c (mmol/l)**	2.2 (1.5, 3.3)	2.2 (1.7, 3.4)	2.7 (2.0, 3.3)		0.320	2.0 (1.6, 2.8)	2.6 (2.1, 3.2)	2.6 (1.9, 3.5)		0.099
** NT-proBNP (pg/dl)**	156.0 (39.7, 360.0)	69.5 (22.3, 281.9)	114.0 (44.5, 496.8)		0.764	117.5 (56.0, 476.0)	121.0 (54.0, 476.5)	158.0 (58.0, 422.5)		0.969
Echocardiography										
** Left atrial diameter (mm)**	35.0 (32.5, 37.1)	36.7 (32.8, 37.9)	36.9 (34.5, 39.4)		0.058	34.2 (32.0, 36.0)	34.9 (33.2, 38.0)	37.0 (35.0, 40.0)		<0.001
** Left atrial area (cm^2^)**	18.5 (16.5, 20.5)	18.0 (16.3, 21.0)	20.0 (17.5, 23.0)		0.046	19.0 (15.5, 20.0)	19.0 (17.0, 21.0)	20.8 (19.0, 23.0)		0.001
** LVESD (mm)**	27.8 (26.0, 30.0)	29.8 (27.2, 31.8)	30.0 (28.2, 33.8)		0.004	29.8 (27.1, 35.5)	30.0 (27.0, 35.3)	30.6 (28.5, 34.0)		<0.001
** LVEDD (mm)**	46.4 ± 4.7	49.2 ± 4.6	50.2 ± 5.0		0.013	42.8 ± 14.2	49.3 ± 4.6	50.5 ± 5.5		<0.001
** LVMI (g/cm^2^)**	70.6 (56.3, 86.0)	69.2 (58.9, 81.9)	65.8 (58.9, 80.4)		0.662	71.4 (59.3, 81.2)	71.0 (61.9, 88.3)	71.9 (63.1, 82.7)		0.569
** LVEF (%)**	70.0 (66.5, 72.0)	69.0 (66.3, 72.0)	69.0 (64.0, 71.8)		0.252	66.0 (53.0, 70.5)	68.0 (62.0, 73.0)	69.5 (64.0, 72.0)		0.324
** E/A**	0.8 (0.7, 0.9)	0.8 (0.7, 1.1)	0.8 (0.7, 1.0)		0.749	0.9 (0.7, 1.1)	0.9 (0.7, 1.1)	0.9 (0.7, 1.2)		0.602
** E/Em**	8.0 (7.5, 9.5)	6.8 (5.3, 9.0)	7.0 (5.0, 8.0)		0.052	7.0 (6.0, 8.5)	6.0 (5.0,8.0)	7.0 (6.0, 9.0)		0.086

Normally distributed data are expressed as the mean ± standard deviation, and nonnormally distributed data are expressed as the median (interquartile range).

CHD, coronary heart disease; CKD, chronic kidney disease; ACEI, angiotensin converting enzyme inhibitor; ARB, angiotensin receptor blocker; ARNI, angiotensin receptor-neprilysin inhibitor; SGLT2i, sodium-glucose cotransporter 2 inhibitors; CCBs, calcium channel blockers; FBG, fasting blood glucose,LDL-c, low-density lipoprotein cholesterol; LVESD, left ventricular end-systolic diameter; LVEDD, left ventricular end-diastolic diameter; LVMI, left ventricular mass index; LVEF, left ventricular ejection fraction.

### 3.2 Changes during the follow-up

#### 3.2.1 Weight loss

During the follow-up period, patients in the GLP-1RA group experienced a significant decrease in weight (6^th^ month vs. Baseline: 80.4 ± 17.3 vs. 84.1 ± 20.1, p<0.001). However, subgroup analysis revealed that the weight improvement was primarily seen in the overweight group (6^th^ month vs. Baseline: 70.0 ± 9.1 vs. 73.1 ± 8.2, p = 0.003) and the obesity group (6^th^ month vs. Baseline: 90.5 ± 14.3 vs. 95.5 ± 16.6, p<0.001). In contrast, no significant change in weight was observed in the normal weight group (6^th^ month vs. Baseline: 57.4 ± 4.8 vs. 58.7 ± 9.2, p=0.063).

In the control group, there was an overall significant decrease in weight (80.6 ± 15.7 vs. 81.4 ± 15.5, p = 0.009). However, this decrease was primarily concentrated in the obesity group (6^th^ month vs. Baseline: 90.4 ± 13.5 vs. 92.7 ± 13.5, p<0.001), resulting in a modest weight loss equivalent to 2.5% of the initial body weight.

#### 3.2.2 Anti-hyperglycemia

In the GLP-1RA group, both fasting blood glucose (FBG) (6^th^ month vs. Baseline: 6.6 (5.7, 8.1) vs. 7.2 (6.2, 10.0), p < 0.001) and HbA1c (6^th^ month vs. Baseline: 6.8 (6.1, 7.4) vs. 7.5 (6.6, 8.9), p < 0.001) showed significant improvement during the follow-up period. Subgroup analysis based on BMI levels revealed that HbA1c significantly improved during the follow-up period, regardless of BMI level. However, the improvement in FBG was not significant in the normal weight group. It is important to note that these improvements in blood glucose levels were not observed in the control group.

#### 3.2.3 Lipid-lowering effect

The lipid-lowering effects of GLP-1RAs vary among patients in different BMI subgroups. In the normal weight group, GLP-1RA does not show improvement in blood lipids. In the overweight group, GLP-1RA improves low density lipoprotein cholesterol(LDL-C) levels (6^th^ month vs. Baseline: 2.2 (1.7, 3.4) vs. 2.0(1.4, 2.5), p=0.012). In the obesity group, after six months of GLP-1RA treatment, total cholesterol (TC, 6^th^ month vs. Baseline: 4.3 (3.6, 4.9) vs. 3.5 (3.0, 4.5), p < 0.001), high density lipoprotein cholesterol(HDL-C, 6^th^ month vs. Baseline: 1.0 (0.8, 1.1) vs.1.0 (0.9, 1.1), p = 0.015), LDL-C (6^th^ month vs. Baseline: 2.6 (2.0, 3.3) vs. 2.1 (1.4, 2.9), p = 0.001), and triglyceride(TG, 6^th^ month vs. Baseline: 2.0 (1.5, 2.8) vs. 1.6 (1.0, 2.2), p = 0.001) levels all significantly improve. While in the control group, only improvements in TC and LDL-C were observed in the overweight and obesity groups.

#### 3.2.4 Changes in cardiac structural and functional parameters

In the GLP-1RA group, only the left ventricular mass index (LVMI) showed a significant improvement during the follow-up period, with values of 63.8 (56.7, 72.3) vs. 67.3 (58.8, 81.3) (p=0.025). None of the other parameters, including LAD, LAA, LVEDD, left ventricular end-systolic diameter (LVESD), left ventricular ejection fraction (LVEF), E/A ratio, and E/Em ratio, exhibited significant changes. However, subgroup analysis revealed significant improvements in LAD (33.0 (30.3, 35.5) vs. 35.0 (32.5, 37.1), p = 0.018) and LAA (18.0 (16.0, 21.5) vs.18.5 (16.5, 20.5), p = 0.008) within the normal weight group. Furthermore, the obesity group exhibited significant improvements in LVEDD (49.8 ± 5.8 vs. 50.2 ± 5.0, p < 0.001) and LVMI (65.1 (56.2, 71.4) vs. 65.8 (58.9, 80.4), p < 0.039). No significant changes were observed in the overweight group.

In contrast, the control group did not show any significant differences in cardiac structural and functional parameters. Subgroup analysis only identified a significant increase in LAD in the normal weight group (35.0 (33.7, 37.3) vs. 34.2 (32.0, 36.0), p = 0.012). No significant changes were observed in the other subgroups during the follow-up (refer to **Tables 3**–**5** for detailed information).

**Table 3:** Changes during the follow-up.

	GLP-1RA Group (N=140)				Control Group (N=140)			
	Baseline	Followup-3M	Followup-6M	P Value	Baseline	Followup-3M	Followup-6M	P Value
General Condition								
**SBP (mmHg)**	135.2 ± 14.5	130.6 ± 13.5	129.2 ± 15.4	<0.001	134.0 ± 16.1	129.0 ± 18.9	128.7 ± 14.3	0.009
**DBP (mmHg)**	79.0 (70.3, 87.0)	80.0 (72.0, 86.0)	76.0 (68.3, 83.8)	0.029	77.0 (69.0, 84.8)	72.0 (66.3, 81.5)	73.0 (67.0,80.0)	0.051
**Heart rate (bpm)**	79.0 (70.0, 88.8)	84.0 (73.0, 91.0)	82.0 (70.0, 87.8)	0.002	77.0 (67.3, 84.8)	76.0 (68.0, 80.0)	72.0 (66.0,80.0)	0.542
**Weight (kg)**	84.1 ± 20.1	82.3 ± 18.0	80.4 ± 17.3	<0.001	81.4 ± 15.5	82.2 ± 15.6	80.6 ± 15.7	0.009
Baseline Biomakers (Serum sample)								
**FPG (mmol/L)**	7.2 (6.2, 10.0)	7.2 (6.0,8.2)	6.6 (5.7, 8.1)	<0.001	6.0 (5.2, 7.5)	6 (5.4, 6.9)	5.9 (5.4,6.8)	0.111
**HbA1c (%)**	7.5 (6.6, 8.9)	7.0 (6.50,7.775)	6.8 (6.1, 7.4)	<0.001	6.2 (5.7, 7.3)	6.5 (6.1, 7.0)	6.4 (5.9,6.9)	0.318
**Uric acid (umol/L)**	369.8 ± 103.3	335.3 ± 100.3	352.2 ± 98.2	0.196	360.2 ± 102.8	356.1 ± 92.3	354.6 ± 87.9	0.908
**TC (mmol/L)**	4.2 (3.4,5.0)	3.3 (2.8,4.1)	3.5 (3.0,4.4)	<0.001	4.1 (3.4,5.0)	3.3 (2.8,4.0)	3.2 (2.7,3.9)	<0.001
**TG (mmol/L)**	1.8 (1.2,2.4)	1.5 (1.0,2.0)	1.5 (1.1, 2.0)	0.001	1.4 (1.1,2.0)	1.3 (1.0,1.7)	1.2 (0.9,1.8)	<0.001
**HDL-c (mmol/L)**	1.0 (0.9, 1.2)	1.0 (0.9,1.2)	1.0 (0.9,1.2)	0.038	1.0 (0.8, 1.2)	1.0 (0.9,1.2)	1.0 (0.9,1.4)	0.012
**LDL-c (mmol/l)**	2.5 (1.8, 3.3)	1.9 (1.5,2.4)	2.0 (1.4,2.6)	<0.001	2.5 (1.9, 3.1)	2.0 (1.4,2.6)	1.7 (1.5,2.3)	<0.001
Echocardiography								
**LAD (mm)**	36.2 (34.0, 38.8)	–	35.0 (33.0,38.0)	0.280	36.0 (33.2, 38.9)	–	37.0 (34.4, 39.5)	0.081
**LAA (cm2)**	19.0 (17.0,22.0)	–	19.5 (18.0,22.0)	0.069	20.0 (18.2, 22.4)	–	20.1 (17.6, 22.8)	0.058
**LVESD (mm)**	30.0 (28.0, 32.0)	–	29.0 (27.8, 31.0)	0.294	30.0 (28.0, 34.0)	–	30.0 (29.0, 33.0)	0.370
**LVEDD (mm)**	49.5 ± 4.8	–	48.5 ± 5.3	0.113	48.9 ± 7.6	–	48.4 ± 9.1	0.076
**LVMI (g/cm^2^)**	67.3 (58.8,81.3)	–	63.8 (56.7,72.3)	0.025	70.9 (61.9, 81.0)	–	69.5 (60.0, 80.3)	0.348
**LVEF (%)**	69.0 (65.0,72.0)	–	68.0 (64.0,72.0)	1.000	68.0 (63.0, 72.0)	–	69.0 (61.0, 72.0)	0.647
**E/A**	0.8 (0.7,1.0)	–	0.8 (0.7,1.1)	0.718	0.9 (0.7, 1.1)	–	0.8 (0.7, 1.0)	0.164
**E/Em**	7.0 (6.0,8.0)	–	6.0 (6.0,8.0)	0.053	7.0 (6.0, 8.0)	–	7.0 (5.0, 8.0)	0.051

Normally distributed data are expressed as the mean ± standard deviation, and nonnormally distributed data are expressed as the median (interquartile range).

SBP, systolic blood pressure; DBP, diastolic blood pressure; FBG, fasting blood glucose; TC, total cholesterol; TG, triglyceride; HDL-c, high-density lipoprotein cholesterol; LDL-c, low-density lipoprotein cholesterol; LAD, left atrial diameter; LAA, left atrial area; LVESD, left ventricular end-systolic diameter; LVEDD, left ventricular end-diastolic diameter; LVMI, left ventricular mass index; LVEF, left ventricular ejection fraction.

**Table 4:** Changes during the follow-up in patients with different BMI in the GLP-1RA group.

	BMI<24kg/m2 (N=22)				BMI 24-28kg/m2 (N=35)				BMI≥28kg/m2 (N=83)			
	Baseline	Followup-3M	Followup-6M	P Value	Baseline	Followup-3M	Followup-6M	P Value	Baseline	Followup-3M	Followup-6M	P Value
General Condition												
**SBP (mmHg)**	131.0 ± 15.0	128.0 ± 16.3	129.8 ± 17.9	0.457	134.1 ± 13.2	131.0 ± 16.2	126.2 ± 15.2	0.007	136.6 ± 14.8	130.9 ± 11.5	130.6 ± 15.1	0.017
**DBP (mmHg)**	70.5 (63.8,79.5)	70.0 (64.0,80.0)	70.0 (57.0,73.0)	0.028	78.5 (69.3,86.3)	75.0 (70.0,80.0)	75.0 (68.0,84.0)	0.175	80.0 (73.0,88.3)	81.5 (76.3,87.0)	80.0 (71.0,85.3)	0.610
**Heart rate (bpm)**	74.5 (68.3,80.0)	82.0 (71.0,85.0)	83.0 (66.0,89.0)	0.075	78.5 (68.0,88.5)	82.5 (68.8,89.0)	80.0 (70.0,91.0)	0.350	79.0 (79.0,90.5)	86.5 (77.5,92.0)	81.6 (70.8,87.0)	0.016
**Weight (kg)**	58.7 ± 9.2	59.0 ± 8.8	57.4 ± 4.8	0.063	73.1 ± 8.2	69.9 ± 8.5	70.0 ± 9.1	0.003	95.5 ± 16.6	93.1 ± 14.3	90.5 ± 14.3	<0.001
Baseline Biomakers (Serum sample)												
**FPG (mmol/L)**	7.1 (6.0,9.6)	6.8 (5.3,11.1)	7.7 (6.1,9.3)	0.262	7.1 (6.7,9.9)	6.7 (6.0,7.3)	6.7 (5.7,8.0)	0.032	7.8 (6.3,11.4)	6.8 (5.4,8.2)	6.7 (5.7,8.0)	<0.001
**HbA1c (%)**	8.3 (6.6,10.2)	8.2 (7.5,10.2)	7.6 (6.9,8.8)	0.015	7.2 (6.9,7.9)	6.8 (6.5,7.1)	6.6 (6.1,7.5)	0.011	7.6 (6.5,8.9)	7.1 (6.4,7.8)	6.6 (5.8,7.2)	<0.001
**Uric acid (umol/L)**	329.2 ± 75.7	280.8 ± 85.3	335.5 ± 100.5	0.010	359.9 ± 92.8	328.4 ± 62.7	337.0 ± 87.5	0.191	384.7 ± 111.3	352.9 ± 113.5	364.9 ± 102.9	0.950
**TC (mmol/L)**	4.1 (3.0, 5.0)	3.5 (2.9,4.1)	3.2 (2.7,4.7)	0.905	4.0 (3.3,5.1)	3.6 (2.9,4.3)	3.7 (3.0,4.3)	0.414	4.3 (3.6,4.9)	3.3 (2.6,4.0)	3.5 (3.0,4.5)	<0.001
**TG (mmol/L)**	1.3 (0.8, 1.9)	0.9 (0.7,1.7)	1.0 (0.8, 1.7)	0.67	1.5 (1.0,2.3)	1.4 (0.9,1.7)	1.4 (0.9, 2.0)	0.161	2.0 (1.5,2.8)	1.5 (1.1,2.1)	1.6 (1.2, 2.2)	0.001
**HDL-c (mmol/L)**	1.1 (0.9,1.4)	1.3 (0.9,1.4)	1.2 (0.9,1.4)	0.836	1.1 (1.0,1.2)	1.1 (0.9,1.2)	1.1 (1.0,1.2)	0.707	1.0 (0.8,1.1)	0.9 (0.8,1.1)	1.0 (0.9,1.1)	0.015
**LDL-c (mmol/l)**	2.2 (1.5,3.2)	1.9 (1.6,2.3)	1.5 (1.3, 2.5)	0.199	2.2 (1.7,3.4)	1.9 (1.6,2.6)	2.0 (1.4, 2.5)	0.012	2.6 (2.0,3.3)	1.9 (1.5,2.6)	2.1 (1.4,2.9)	0.001
Echocardiography												
**LAD (mm)**	35.0 (32.5,37.1)	–	33.0 (30.3,35.5)	0.018	36.7 (32.8,37.9)	–	33.0 (31.8,35.3)	0.170	36.9 (34.5,39.4)		37.0 (35.0,39.0)	0.342
**LAA (cm2)**	18.5 (16.5, 20.5)	–	18.0 (16.0, 21.5)	0.008	18.0 (16.3,21.0)	–	18.0 (16.3, 20.6)	0.739	20.0 (17.5,23.0)		21.0 (18.3, 22.4)	0.067
**LVESD (mm)**	27.8 (26.0,30.0)	–	28.0 (26.2, 29.3)	0.477	29.8 (27.2,31.8)	–	29.0 (28.0, 31.1)	0.776	30.0 (28.2,33.8)		29.0 (27.8, 31.5)	0.405
**LVEDD (mm)**	46.4 ± 4.7	–	44.7 ± 2.9	0.095	49.2 ± 4.6	–	44.7 ± 2.9	0.246	50.2 ± 5.0	–	49.8 ± 5.8	<0.001
**LVMI (g/cm2)**	70.6 (56.3,86.0)	–	60.9 (53.3,77.0)	0.173	69.2 (58.9,81.9)	–	62.3 (58.3,77.1)	0.647	65.8 (58.9,80.4)		65.1 (56.2,71.4)	0.039
**LVEF (%)**	70.0 (66.5, 72.0)	–	69.0 (65.0,71.5)	0.482	69.0 (66.3,72.0)	–	68.0 (64.0,72.0)	0.468	69.0 (64.0,71.8)		68.0 (63.0,72.0)	0.325
**E/A**	0.8 (0.7, 0.9)	–	0.8 (0.6, 0.9)	0.086	0.8 (0.7,1.1)	–	0.8 (0.7,1.0)	0.841	0.8 (0.7,1.0)		0.9 (0.7,1.1)	0.153
**E/Em**	8.0 (7.5,9.5)	–	6.0 (6.0,9.0)	0.072	6.8 (5.3,9.0)	–	6.0 (5.0,7.0)	<0.001	7.0 (5.0,8.0)		7.0 (6.0,7.0)	0.88

Normally distributed data are expressed as the mean ± standard deviation, and nonnormally distributed data are expressed as the median (interquartile range).

SBP, systolic blood pressure; DBP, diastolic blood pressue; FBG, fasting blood glucose; TC, total cholesterol; TG, triglyceride; HDL-c, high-density lipoprotein cholesterol; LDL-c, low-density lipoprotein cholesterol; LAD, left atrial diameter; LAA, left atrial area; LVESD, left ventricular end-systolic diameter; LVEDD, left ventricular end-diastolic diameter; LVMI, left ventricular mass index; LVEF, left ventricular ejection fraction.

**Table 5:** Changes during the follow-up in patients with different BMI in the control group.

	BMI<24kg/m2 (N=22)				BMI 24-28kg/m2 (N=51)				BMI≥28kg/m2 (N=67)			
	Baseline	Followup-3M	Followup-6M	P Value	Baseline	Followup-3M	Followup-6M	P Value	Baseline	Followup-3M	Followup-6M	P Value
General Condition												
**SBP (mmHg)**	133.3 ± 15.9	130.5 ± 21.6	129.8 ± 10.0	0.410	130.0 ± 14.6	125.1 ± 22.7	120.0 ± 10.7	0.028	136.6 ± 16.9	132.4 ± 11.1	133.3 ± 15.2	0.091
**DBP (mmHg)**	69.5 (63.0,79.0)	68.5 (65.8,82.8)	68.0 (62.0,81.0)	0.867	75.5 (70.3,81.0)	71.5 (65.0,78.5)	71.0 (65.8,76.3)	0.103	78.5 (70.3,90.0)	76.5 (70.0,85.0)	75.0 (70.0,80.0)	0.108
**Heart rate (bpm)**	73.5 (61.8,84.5)	76.0 (69.8,82.8)	74.0 (69.0,80.0)	0.290	77.5 (68.0,86.5)	76.5 (68.0,85.3)	72.0 (64.3,81.8)	0.878	77.5 (69.5,84.3)	73.5 (67.0,79.8)	72.0 (66.0,80.0)	0.340
**Weight (kg)**	64.2 ± 6.7	65.8 ± 7.4	63.9 ± 7.8	0.576	73.8 ± 6.7	75.1 ± 6.9	73.5 ± 8.2	0.834	92.7 ± 13.5	91.5 ± 13.3	90.4 ± 13.5	0.008
Baseline Biomakers (Serum sample)												
**FPG (mmol/L)**	5.8 (5.0,9.0)	6.0 (5.3,8.5)	6.2 (5.2,7.8)	0.030	5.7 (5.0,7.9)	6.0 (5.5,7.2)	5.7 (5.1,6.7)	0.972	6.1 (5.4,7.4)	6.0 (5.3,6.9)	5.9 (5.4,6.7)	0.322
**HbA1c (%)**	6.2 (5.8,7.1)	6.5 (6.3,6.7)	6.5 (5.8,6.8)	0.368	6.2 (5.7,7.3)	6.6 (5.8,7.5)	6.5 (5.9,7.2)	0.469	6.2 (5.8,7.2)	6.5 (6.1,7.0)	6.4 (5.9,7.0)	0.302
**Uric acid (umol/L)**	309.8 ± 78.6	309.9 ± 70.1	314.1 ± 60.9	0.500	349.4 ± 90.9	347.1 ± 90.7	349.2 ± 93.8	0.969	384.0 ± 111.8	376.0 ± 94.6	369.2 ± 87.9	0.776
**TC (mmol/L)**	3.9 (3.1, 4.8)	3.0 (2.6,3.6)	3.1 (2.7,3.5)	0.167	4.1 (3.5,5.2)	3.2 (2.8,4.1)	3.0 (2.6,3.5)	<0.001	4.1 (3.3,5.0)	3.5 (2.7,4.0)	3.5 (2.9,4.3)	0.006
**TG (mmol/L)**	1.1 (0.9,1.4)	1.5 (0.8,1.9)	1.1 (0.8,1.7)	0.386	1.5 (1.1,2.0)	1.3 (0.8,1.8)	1.0 (0.9,1.6)	0.051	1.4 (1.1,2.2)	1.5 (1.8, 2.5)	1.3 (1.0,1.9)	0.120
**HDL-c (mmol/L)**	1.1 (0.8,1.2)	1.2 (1.0, 1.6)	1.1 (1.0,1.3)	0.453	1.0 (0.8,1.1)	1.2 (1.0,1.7)	1.0 (0.9,1.2)	0.836	1.0 (0.8,1.1)	1.3 (1.0,1.8)	1.0 (0.9,1.2)	0.077
**LDL-c (mmol/l)**	2.0 (1.6,2.8)	1.0 (0.9,1.3)	1.6 (1.5,1.9)	0.366	2.6 (2.1,3.2)	1.0 (0.9,1.3)	1.7 (1.5,1.9)	<0.001	2.6 (1.9,3.5)	1.0 (0.8,1.2)	1.8 (1.5,2.6)	<0.001
Echocardiography												
**LAD (mm)**	34.2 (32.0,36.0)	–	35.0 (33.7,37.3)	0.012	34.9 (33.2,38.0)	–	35.5 (33.6,39.0)	0.318	37.0 (35.0,40.0)	–	38.0 (35.2 40.9)	0.952
**LAA (cm2)**	19.0 (15.5,20.0)	–	19.0 (16.0,21.0)	0.161	19.0 (17.0,21.0)	–	20.0 (18.0,21.8)	0.238	20.8 (19.0,23.0)	–	21.0 (19.0,23.8)	0.426
**LVESD (mm)**	29.8 (27.1,35.5)	–	29.8 (27.5,33.0)	0.683	30.0 (27.0,35.3)	–	30.0 (28.1,32.7)	0.857	30.6 (28.5,34.0)	–	30.0 (29.2,33.0)	0.216
**LVEDD (mm)**	42.8 ± 14.2	–	44.7 ± 11.1	0.822	49.3 ± 4.6	–	48.0 ± 8.7	0.536	50.5 ± 5.5	–	50.0 ± 8.4	0.231
**LVMI (g/cm2)**	71.4 (59.3, 81.2)	–	61.5 (48.8, 72.7)	0.124	71.0 (61.9, 88.3)	–	70.0 (59.7, 81.6)	0.820	71.9 (63.1, 82.7)	–	71.0 (60.5, 81.6)	0.724
**LVEF (%)**	66.0 (53.0, 70.5)	–	63.5 (52.0, 72.0)	0.729	68.0 (62.0, 73.0)	–	69.0 (60.5, 72.5)	0.741	69.5 (64.0, 72.0)	–	69.0 (60.5, 71.5)	0.470
**E/A**	0.9 (0.7, 1.1)	–	0.8 (0.6, 1.1)	0.470	0.9 (0.7, 1.1)	–	0.8 (0.7, 1.3)	0.292	0.9 (0.7, 1.2)	–	0.8 (0.7, 1.0)	0.567
**E/Em**	7.0 (6.0, 8.5)	–	6.5 (5.0, 9.0)	0.439	6.0 (5.0,8.0)	–	6.0 (5.0, 8.0)	0.479	7.0 (6.0, 9.0)	–	7.0 (5.0, 8.0)	0.060

Normally distributed data are expressed as the mean ± standard deviation, and nonnormally distributed data are expressed as the median (interquartile range).

SBP, systolic blood pressure; DBP, diastolic blood pressue; FBG, fasting blood glucose; TC, total cholesterol; TG, triglyceride; HDL-c, high-density lipoprotein cholesterol; LDL-c, low-density lipoprotein cholesterol; LAD, left atrial diameter; LAA, left atrial area; LVESD, left ventricular end-systolic diameter; LVEDD, left ventricular end-diastolic diameter; LVMI, left ventricular mass index; LVEF, left ventricular ejection fraction.

#### 3.2.5 Effects of GLP-1RA application on cardiac structure

As stated earlier, the alterations in echocardiographic parameters predominantly pertained to cardiac structure throughout the follow-up period. Consequently, we conducted univariate and multivariate analyses in subgroup studies to investigate the influence of GLP-1RA administration on cardiac structural parameters using generalized estimating equations. The multivariate analysis encompassed factors that could potentially affect the outcomes, including age, systolic blood pressure, diastolic blood pressure, gender, history of diabetes, hyperlipidemia, CHD, stroke, utilization of statins, SGLT2i, and baseline FBG levels.

In the normal weight group, the univariate analysis did not show any significant impact of grouping (GLP-1RA group and control group) on the changes in LAD, LAA, LVEDD, or LVMI. However, the multivariate analysis demonstrated that grouping could independently influence the changes in LAD (p = 0.013) and LAA (p = 0.008) during the follow-up period. In the obesity group, both the univariate and multivariate analyses indicated that grouping had an effect on the changes in LVMI during follow-up, but no significant effect was observed on the changes in other parameters. Conversely, no influence of grouping on cardiac structural parameters was observed in the overweight group (refer to **Table 6** for detailed information).

**Table 6:** Univariate and multivariate analyses of the changes of cardiac structure indexes among the groups during the follow-up.

	BMI<24kg/m2				BMI 24-28kg/m2				BMI≥28kg/m2			
	GLP-1RA	Control	P1 Value	P2 Value	GLP-1RA	Control	P1 Value	P2 Value	GLP-1RA	Control	P1 Value	P2 Value
**Left atrial diameter-BL (mm)**	34.8 (33.4,36.3)	34.5 (33.3,35.7)	0.730	0.013	35.7 (34.5,37.0)	35.1 (34.1,36.0)	0.432	0.909	37.3 (36.4,38.1)	37.7 (36.6,38.7)	0.581	0.855
**Left atrial diameter-6M (mm)**	32.0 (29.5,34.6)	36.0 (34.7,37.3)			34.7 (33.4,36.0)	35.9 (34.7,37.0)			37.8 (36.5,39.0)	38.5 (37.1,39.9)		
**P Value**	0.004	0.006			0.053	0.160			0.349	0.150		
**Left atrial area-BL (cm2)**	18.7 (16.4,20.9)	18.5 (17.2,19.8)	0.541	0.008	18.8 (17.6,20.0)	19.4 (18.5,20.3)	0.438	0.379	20.2 (19.2,21.2)	21.2 (20.1,22.3)	0.257	0.273
**Left atrial area-6M (cm2)**	18.3 (17.0,19.6)	20.3 (17.1,23.5)			18.7 (17.6,19.8)	20.8 (19.2,22.4)			21.6 (20.7,22.5)	21.9 (20.5,23.3)		
**P Value**	0.694	0.228			0.829	0.123			0.026	0.202		
**LVEDD-BL (mm)**	46.4 (44.3,48.5)	42.8 (36.7,48.8)	0.289	0.760	49.3 (47.7,50.9)	49.1 (47.8,50.4)	0.935	0.620	50.2 (49.3,52.0)	50.5 (49.3,52.0)	0.922	0.934
**LVEDD-6M (mm)**	44.7 (42.9,46.4)	44.7 (39.2,50.3)			48.3 (46.6,49.9)	48.0 (44.9,51.0)			49.8 (48.4,51.5)	50.0 (46.9,52.1)		
**P Value**	0.190	0.660			0.086	0.488			0.614	0.385		
**LVMI-BL (g/cm2)**	74.3 (63.5,85.1)	69.9 (64.2,75.8)	0.430	0.193	77.1 (65.9,88.3)	74.5 (67.1,82.0)	0.821	0.144	69.7 (66.4,72.9)	76.4 (71.9,80.8)	0.007	0.011
**LVMI-6M (g/cm2)**	65.7 (58.1,73.4)	61.6 (55.4,67.7)			73.2 (63.8,82.6)	72.4 (65.3,79.6)			65.5 (61.9,69.1)	73.2 (67.0,79.5)		
**P Value**	0.051	0.033			0.409	0.677			0.045	0.246		

Normally distributed data are expressed as the mean ± standard deviation, and nonnormally distributed data are expressed as the median (interquartile range).

BL, baseline; 6M, 6^th^ month of the follow-up; LVEDD, left ventricular end-diastolic diameter; LVMI, left ventricular mass index.

P value: Changes in this indicator during the follow-up period, P1 value: Univariate analysis of the impact of grouping on changes in this indicator, P2 value: Multivariate analysis of the impact of grouping on changes in this indicator, The multivariate analysis encompassed factors that could potentially affect the outcomes, including age, systolic blood pressure, diastolic blood pressure, gender, history of diabetes, hyperlipidemia, coronary heart disease, stroke, utilization of statins, SGLT2 inhibitors, and fasting blood glucose levels.

## 4 Discussion

GLP-1 is a hormone secreted by the small intestine that reduces blood glucose levels by stimulating insulin production and inhibiting glucagon secretion. GLP-1RA is a novel hypoglycemic medication recommended as a first-line therapy for patients with diabetes who are at high cardiovascular risk or have established cardiovascular disease (9). However, the majority of subjects included in the existing clinical evidence are obese individuals (3, 4, 6, 7). It remains unclear whether GLP-1RA would yield the same cardiovascular benefits in individuals with normal or overweight weight. Moreover, in individuals with normal weight or underweight, the level of cardiovascular risk is inversely proportional to BMI (8). Therefore, it is crucial to investigate how drugs can be utilized to further enhance the cardiac prognosis of such patients.

In this study, we enrolled patients diagnosed with ASCVD who were taking GLP-1RA, and we included age- and BMI-matched controls. The subjects were followed up for 6 months to assess changes in body weight, metabolic parameters, and cardiac structure and function. Subgroup analysis was conducted based on baseline BMI levels. The key findings of this study are as follows:

1. In the study, it was found that GLP-1RA only led to a reduction in body weight among overweight and obese patients, while it did not have any effect on body weight in normal-weight individuals.
2. In the study, it was observed that GLP-1RA improved blood glucose levels irrespective of BMI levels; however, the improvement in blood lipid metabolism was more pronounced in patients with obesity.
3. In the study, the effects of GLP-1RA on cardiac structure were found to vary among patients with different baseline BMI levels. Specifically, it was observed that the improvement in atrial structure was more significant in patients with normal body weight, whereas the improvement in ventricular structure was more prominent in obese subjects.

### 4.1 Effect of GLP-1RA on body weight

GLP-1RA has been shown to have significant efficacy in weight reduction. Numerous meta-analyses and large-scale randomized controlled trials (RCTs) have confirmed the weight-reducing effects of GLP-1RA, regardless of whether patients have diabetes or not (10). Furthermore, the weight reduction effect becomes more pronounced with increasing baseline BMI (6, 7, 11, 12). The FDA has approved liraglutide 3mg once daily and semaglutide 2.4mg once weekly for weight loss treatment (13). However, as previously mentioned, the populations included in these studies mostly consist of individuals with obesity, with a smaller proportion of individuals with normal or low BMI, which is of particular interest to us. For these populations, who often have longer durations of diabetes, higher baseline HbA1c levels, and poorer β-cell function, more targeted research results are needed to provide a basis for individualized treatment (10, 14). In addition, although a limited number of *post-hoc* analyses suggest that GLP-1RA still has a weight-reducing effect in normal or underweight individuals, these studies have not adequately controlled for various confounding factors, which may affect the reliability of the results (10–12). Therefore, to further clarify the impact of GLP-1RA on weight across the entire BMI range, this study conducted subgroup analyses based on baseline BMI levels. The findings revealed significant weight reduction effects of GLP-1RA in overweight or obese individuals, with the most pronounced effects observed in the obese population. However, for individuals with normal or low BMI, GLP-1RA did not significantly affect patients’ weight, in this population, weight reduction did not provide additional benefits and may even increase the risk of long-term cardiovascular disease. Hence, these results also provide some evidence regarding the safety of GLP-1RA in this patient population, but larger-scale RCTs are needed to confirm these findings and establish stronger evidence.

### 4.2 Effect of GLP-1RA on metabolism

Abnormal metabolic parameters, including blood glucose and lipid levels, are significant risk factors for atherosclerosis. The anti-atherosclerotic effect of GLP-1RAs may be partially attributed to their ability to improve these metabolic parameters (7, 11, 15–17). Regarding the effects of GLP-1RA on blood glucose control, consistent with previous research (7, 11, 15), GLP-1RA demonstrates its glucose-lowering efficacy regardless of the baseline BMI levels of the patients. However, there are differences in the improvement of blood lipids among patients with different baseline BMIs. In the normal weight group, GLP-1RA does not show improvement in blood lipids. In the overweight group, GLP-1RA improves LDL-C levels. In the obesity group, after six months of GLP-1RA treatment, TC, HDL-C, LDL-C, and TG levels all significantly improve. Therefore, with increasing BMI, the improvement in lipid profile with GLP-1RA becomes more pronounced. Reviewing previous studies, the SUSTAIN and REWIND trials have evaluated the lipid levels of the participants, and the results suggest that GLP-1RA has an improving effect on TC, LDL-C, and TG (3, 7). In a *post-hoc* analysis by John W et al. (18), patients were divided into three groups based on baseline BMI: less than 30 kg/m², 30-35 kg/m², and greater than 35 kg/m². The study found that exenatide significantly reduces LDL-C levels in the latter two groups, but it has no significant impact on LDL-C in patients with a BMI less than 30 kg/m², which is consistent with the findings of this study. The mechanism behind the improvement of lipid profile with GLP-1RA has not been fully elucidated. It may be related to the improvement in blood glucose, reduction in insulin resistance, stimulation of hepatic triglyceride production leading to decreased serum TG levels, as well as a possible reduction in the secretion of intestinal chylomicrons through GLP-1 receptors on the intestinal mucosa, thus reducing TG absorption. Furthermore, Li et al. found that the improvement in lipid profile associated with liraglutide may be achieved through the regulation of gene expression (possibly Acrp30) and protein expression (19).

### 4.3 Effect of GLP-1RA on cardiac structure

According to the findings of this study, GLP-1RA treatment for 6 months has different effects on cardiac structure in subgroups with different BMI. In the normal or low BMI group, GLP-1RA significantly improves atrial structure, while in the obesity group, it improves ventricular structure. No significant impact of this medication on cardiac function was observed. Previous studies have indicated that obesity is associated with cardiac remodeling, manifested by enlargement of left atrial and ventricular dimensions and an increase in left ventricular mass (20–22). Animal experiments and network meta-analyses suggest that the use of GLP-1RA can improve parameters such as LVESD and LVMI (23, 24). Similar improvements in cardiac structure with GLP-1RA were observed in this study, but the effects varied among individuals with different BMI, which has not been explored in previous studies, and the reasons for this difference are not yet fully understood. It is known that GLP-1 receptors are predominantly expressed in the atria, suggesting that GLP-1RA may directly protect the atria by increasing cellular glucose uptake and inhibiting inflammatory stress (25). Ventricular distribution of GLP-1 receptors is less prominent, so the medication may indirectly protect the ventricles through a series of mechanisms (9). Previous studies involving Asian populations have shown an independent correlation between overweight/obesity and the changes in ventricular structural parameters (22). The weight reduction observed after the administration of GLP-1RA can reduce cardiac load through volume and pressure reduction (26). Additionally, weight loss can alleviate endoplasmic reticulum stress-induced myocardial cell apoptosis, resulting in more orderly and denser arrangement of myocardial cells and promoting intracellular Ca^2+^ homeostasis, thereby reducing myocardial autophagy and ventricular remodeling (27). Furthermore, researchers have observed improvement in microcirculation in overweight individuals after the administration of GLP-1RA, which was not observed in individuals with normal or low body weight (28). Microcirculation dysfunction is crucial for ventricular remodeling. Therefore, we hypothesize that, since there was no significant improvement in body weight in the normal or low BMI group in our study, GLP-1RA may attenuate atrial remodeling directly through the GLP-1R, whereas in the other two groups, GLP-1RA may attenuate ventricular remodeling indirectly through significant weight improvement. As for cardiac function, obese patients generally retain normal systolic function, but inflammation, oxidative stress, and disturbances in glucose and lipid metabolism may lead to a decline in diastolic function (24). In this study, the baseline cardiac systolic and diastolic functions of the patients were roughly normal, and no significant changes were observed during the follow-up period. Further investigation of cardiac function may require the use of more sensitive indicators such as speckle tracking or cardiac magnetic resonance.

### 4.4 Strengths and limitations

In order to clarify whether GLP-1RA can exert consistent effects on weight reduction, glycemic control, and improvement of cardiac remodeling in ASCVD patients with different baseline BMIs, we designed and conducted the above-mentioned study. According to the results, we discovered that GLP-1RA does not significantly affect the weight of patients with normal or low BMI, thereby partially confirming the safety of this medication. Further weight reduction in this demographic may potentially result in a higher occurrence of adverse events (8). Additionally, the study found that cardiac remodeling may have disparate effects on patients with different BMIs. While this finding necessitates confirmation through larger-scale studies, it may offer new perspectives and ideas for future research on the application of GLP-1RA. However, this study has the following limitations: 1) It is a single-center retrospective cohort study, and the conclusions need further confirmation through large-scale RCTs in the future. 2) Previous studies have already found significant atrial remodeling in obese patients, which may be associated with oxidative stress, chronic inflammatory, and infiltration of epicardial adipose tissue associated with obesity (29, 30). However, in this study, no improvement in atrial remodeling after weight loss was observed, and the underlying mechanisms for this result need further clarification.

## 5 Conclusion

This study evaluated the impact of GLP-1RA application on cardiac remodeling in ASCVD patients with different BMI. The findings revealed that GLP-1RA can improve atrial remodeling in patients with normal or low BMI. However, in obese patients, GLP-1RA shows improvement in ventricular remodeling. The observed differences in these effects may be associated with its varying impact on body weight. These findings highlight the potential benefits of GLP-1RA in improving specific aspects of cardiac structure depending on the patient’s BMI.

## Data availability statement

The original contributions presented in the study are included in the article/supplementary material. Further inquiries can be directed to the corresponding author.

## Ethics statement

The studies involving humans were approved by Medical Science Research Ethics Committee of Peking university third hospital. The studies were conducted in accordance with the local legislation and institutional requirements. The ethics committee/institutional review board waived the requirement of written informed consent for participation from the participants or the participants’ legal guardians/next of kin because Since this study was a retrospective cohort study that only gathered clinical data from participants, an exemption from informed consent was obtained after a review by the Medical Science Research Ethics Committee.

## Author contributions

LX: Conceptualization, Data curation, Formal analysis, Investigation, Methodology, Writing – original draft, Writing – review & editing. DZ: Conceptualization, Formal analysis, Supervision, Validation, Writing – review & editing.
